# Redetermination of 2,6-dimethoxy­benzoic acid

**DOI:** 10.1107/S1600536809001408

**Published:** 2009-01-17

**Authors:** Gustavo Portalone

**Affiliations:** a Chemistry Department, "Sapienza" University of Rome, P.le A. Moro, 5, I-00185 Rome, Italy

## Abstract

The crystal structure of the title compound, C_9_H_10_O_4_, was first reported by Swaminathan, Vimala & Lotter [*Acta Cryst.* (1976), B**32**, 1897–1900]. It has been re-examined, improving the precision of the derived geometric parameters. The asymmetric unit comprises a non-planar independent mol­ecule, as the meth­oxy substituents force the carb­oxy group to be twisted away from the plane of the aromatic ring by 56.12 (9)°. Due to the anti­planar conformation adopted by the OH group, the mol­ecular components do not form the conventional dimeric units, but are associated in the crystal in chains stabilized by linear O—H⋯O hydrogen bonds, involving the OH groups and the carbonyl O atoms, which form *C*(3) motifs.

## Related literature

For previous structure determinations, see: Swaminathan *et al.* (1976 ▶); Bryan & White (1982 ▶). For related literature, see: Gopalakrishna & Cartz, 1972 ▶; Leiserowitz, 1976 ▶; Byriel *et al.*, 1991 ▶; Chen *et al.*, 2007 ▶. For computation of ring patterns formed by hydrogen bonds in crystal structures, see: Etter *et al.* (1990 ▶); Bernstein *et al.* (1995 ▶); Motherwell *et al.* (1999 ▶). For a description of the Cambridge Structural Database, see: Allen (2002 ▶).
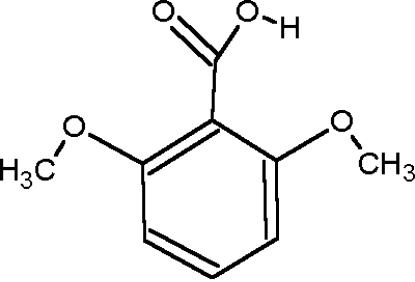


## Experimental

### 

#### Crystal data

C_9_H_10_O_4_
*M* *_r_* = 182.17
Orthorhombic, 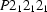

*a* = 7.12255 (13) Å
*b* = 8.92296 (15) Å
*c* = 13.79430 (18) Å
*V* = 876.69 (2) Å^3^
*Z* = 4
Mo *K*α radiation
μ = 0.11 mm^−1^
*T* = 298 (2) K
0.12 × 0.10 × 0.10 mm

#### Data collection

Oxford Diffraction Xcalibur S CCD diffractometer
Absorption correction: multi-scan (*CrysAlis RED*; Oxford Diffraction, 2006 ▶) *T*_min_ = 0.967, *T*_max_ = 0.999
234729 measured reflections
1246 independent reflections
1241 reflections with *I* > 2σ(*I*)
*R*_int_ = 0.039

#### Refinement

*R*[*F* ^2^ > 2σ(*F* ^2^)] = 0.042
*wR*(*F* ^2^) = 0.110
*S* = 1.25
1246 reflections
129 parameters
H atoms treated by a mixture of independent and constrained refinement
Δρ_max_ = 0.17 e Å^−3^
Δρ_min_ = −0.20 e Å^−3^

### 

Data collection: *CrysAlis CCD* (Oxford Diffraction, 2006 ▶); cell refinement: *CrysAlis RED* (Oxford Diffraction, 2006 ▶); data reduction: *CrysAlis RED*; program(s) used to solve structure: *SIR97* (Altomare *et al.*, 1999 ▶); program(s) used to refine structure: *SHELXL97* (Sheldrick, 2008 ▶); molecular graphics: *ORTEP-3* (Farrugia, 1997 ▶); software used to prepare material for publication: *WinGX* (Farrugia, 1999 ▶).

## Supplementary Material

Crystal structure: contains datablocks global, I. DOI: 10.1107/S1600536809001408/kp2203sup1.cif

Structure factors: contains datablocks I. DOI: 10.1107/S1600536809001408/kp2203Isup2.hkl

Additional supplementary materials:  crystallographic information; 3D view; checkCIF report

## Figures and Tables

**Table 1:** Hydrogen-bond geometry (Å, °)

*D*—H⋯*A*	*D*—H	H⋯*A*	*D*⋯*A*	*D*—H⋯*A*
O2—H2⋯O1^i^	0.87 (4)	1.82 (4)	2.681 (2)	172 (4)

Symmetry code: (i) 
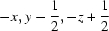
.

**Fig. 1.**
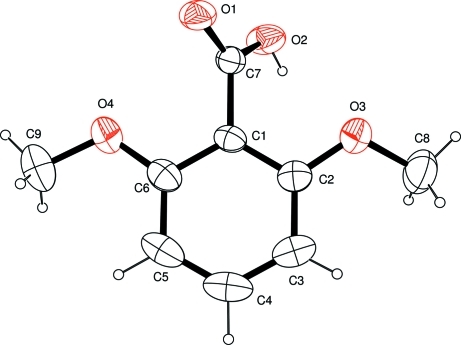
The molecular structure of (I) showing the atom-labelling scheme. Displacements ellipsoids are at the 50% probability level.

**Fig. 2.**
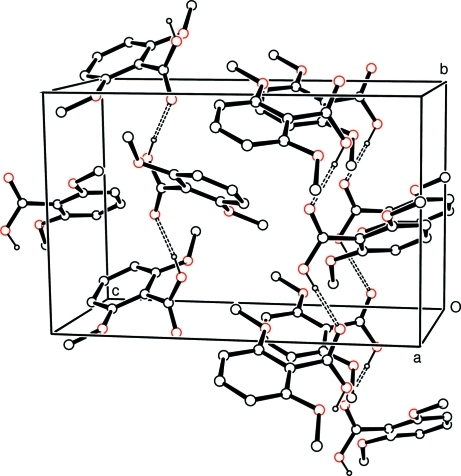
Crystal packing diagram for (I) viewed approximately down b. All atoms are shown as small spheres of arbitrary radii. For the sake of clarity, H atoms not involved in hydrogen bonding have been omitted. Hydrogen bonding is indicated by dashed lines.

## supplementary crystallographic information

### Comment

2,6-Dimethoxybenzoic acid was determined some 30 years ago (Swaminathan *et al.*, 1976) but the final refinement was carried only to *R*=0.15 and no atomic coordinates were provided. Subsequently, a new X-ray structure determination was reported (Bryan & White, 1982). In this study, 775 unique reflections were collected at ambient temperature on an automatic diffractometer using Cu Kα radiation. Data were corrected for Lp effects, but not for absorption [µ(Cu Kα)= 94 mm^-1^]. 708 reflections having values significantly above background were used in the block-diagonal least-squares refinement. The final calculations, carried out on a fairly small data set, led to *R* = 0.035 for 158 refined parameters, a data-to-parameter ratio of 4.5, the maximum shift-to-error in the final cycle being equal to 1/4, and standard deviations of 0.005Å in C—C bond lengths and 0.4° in bond angles.

The asymmetric unit of (I) comprises a non-planar independent molecule, as the methoxy substituents force the carboxy group to be twisted away from the plane of the aromatic ring by 56.12 (9)° (Fig. 1). The values of bond lengths and bond angles are consistent with that reported in the previous determination (Bryan & White, 1982) with the exception of the geometrical parameters of the carboxy group. Analysis of the crystal packing of (I), (Table 1, Fig. 2) shows that the molecular components do not form the conventional dimeric units observed in monocarboylic acids (Leiserowitz, 1976). The structure is stabilized by intermolecular O—H···O interactions of descriptor C(3) (Etter *et al.*, 1990; Bernstein *et al.*, 1995; Motherwell *et al.*, 1999) (Table 1, Fig. 2) between the OH moieties and the carbonyl O atom (O1^i^) [symmetry code: (i) -*x*, *y* - 1/2, -*z* + 1/2] which link the molecules into endless chains approximately parallel to b. A search of the Cambridge Structural Database (version 5.29; Allen, 2002) for crystal structures containing the *o*-methoxy benzoic acid residue yields only three structures having the OH group in the unusual antiplanar conformation (Gopalakrishna & Cartz, 1972; Byriel *et al.*, 1991; Chen *et al.*, 2007). For these compounds, the antiplanar conformation is favoured by the formation of intramolecular hydrogen bonding.

### Experimental

2,6-Dimethoxybenzoic acid (0.1 mmol, Sigma Aldrich at 99% purity) was dissolved in ethanol (95%, 9 mL) and gently heated under reflux for 1 h. After cooling the solution to an ambient temperature, crystals suitable for single-crystal X-ray diffraction were grown by slow evaporation of the solvent after two days.

### Refinement

All H atoms were found in a difference map and then treated as riding atoms, with C—H = 0.97 (phenyl) and 0.96–0.98 Å (methyl); their *U*_iso_ values were kept equal to 1.2*U*_eq_(C, phenyl). and to 1.5*U*_eq_(C, methyl). The remaining H atom of the carboxy group was freely refined. In the absence of significant anomalous scattering in this light-atom study, Friedel pairs were merged.

### Crystal data

**Table:**

C_9_H_10_O_4_	*F*(000) = 384
*M**_r_* = 182.17	*D*_x_ = 1.380 Mg m^−^^3^
Orthorhombic, *P*2_1_2_1_2_1_	Mo *K*α radiation, λ = 0.71073 Å
Hall symbol: P 2ac 2ab	Cell parameters from 75565 reflections
*a* = 7.12255 (13) Å	θ = 2.7–32.6°
*b* = 8.92296 (15) Å	µ = 0.11 mm^−^^1^
*c* = 13.79430 (18) Å	*T* = 298 K
*V* = 876.69 (2) Å^3^	Tablets, colourless
*Z* = 4	0.12 × 0.10 × 0.10 mm

### Data collection

**Table:**

Oxford Diffraction Xcalibur S CCD diffractometer	1246 independent reflections
Radiation source: Enhance (Mo) X-ray Source	1241 reflections with *I* > 2σ(*I*)
graphite	*R*_int_ = 0.039
Detector resolution: 16.0696 pixels mm^-1^	θ_max_ = 28.0°, θ_min_ = 2.7°
ω and φ scans	*h* = −9→9
Absorption correction: multi-scan (*CrysAlis RED*; Oxford Diffraction, 2006)	*k* = −11→11
*T*_min_ = 0.967, *T*_max_ = 0.999	*l* = −18→18
234729 measured reflections	

### Refinement

**Table:**

Refinement on *F*^2^	Primary atom site location: structure-invariant direct methods
Least-squares matrix: full	Secondary atom site location: difference Fourier map
*R*[*F*^2^ > 2σ(*F*^2^)] = 0.042	Hydrogen site location: inferred from neighbouring sites
*wR*(*F*^2^) = 0.110	H atoms treated by a mixture of independent and constrained refinement
*S* = 1.25	*w* = 1/[σ^2^(*F*_o_^2^) + (0.059*P*)^2^ + 0.1016*P*] where *P* = (*F*_o_^2^ + 2*F*_c_^2^)/3
1246 reflections	(Δ/σ)_max_ < 0.001
129 parameters	Δρ_max_ = 0.17 e Å^−^^3^
0 restraints	Δρ_min_ = −0.20 e Å^−^^3^

### Special details

**Table:**

Experimental. CrysAlis RED (Oxford Diffraction, 2008) Version 1.171.32.15 (release 10-01-2008 CrysAlis171 .NET) (compiled Jan 10 2008,16:37:18) Empirical absorption correction using spherical harmonics, implemented in SCALE3 ABSPACK scaling algorithm.
Geometry. All e.s.d.'s (except the e.s.d. in the dihedral angle between two l.s. planes) are estimated using the full covariance matrix. The cell e.s.d.'s are taken into account individually in the estimation of e.s.d.'s in distances, angles and torsion angles; correlations between e.s.d.'s in cell parameters are only used when they are defined by crystal symmetry. An approximate (isotropic) treatment of cell e.s.d.'s is used for estimating e.s.d.'s involving l.s. planes.
Refinement. Refinement of *F*^2^ against ALL reflections. The weighted *R*-factor *wR* and goodness of fit *S* are based on *F*^2^, conventional *R*-factors *R* are based on *F*, with *F* set to zero for negative *F*^2^. The threshold expression of *F*^2^ > σ(*F*^2^) is used only for calculating *R*-factors(gt) *etc*. and is not relevant to the choice of reflections for refinement. *R*-factors based on *F*^2^ are statistically about twice as large as those based on *F*, and *R*- factors based on ALL data will be even larger.

### Fractional atomic coordinates and isotropic or equivalent isotropic displacement parameters (Å^2^)

**Table:**

	*x*	*y*	*z*	*U*_iso_*/*U*_eq_	
O1	−0.0502 (3)	0.05267 (15)	0.21547 (10)	0.0450 (4)	
O2	−0.0480 (3)	−0.18967 (17)	0.19834 (12)	0.0488 (4)	
H2	−0.005 (6)	−0.272 (4)	0.224 (2)	0.080 (11)*	
O3	0.3469 (2)	−0.20799 (17)	0.23289 (11)	0.0456 (4)	
O4	−0.1001 (2)	0.03782 (19)	0.42166 (10)	0.0523 (4)	
C1	0.1298 (3)	−0.08417 (18)	0.33092 (12)	0.0313 (4)	
C2	0.3031 (3)	−0.1561 (2)	0.32308 (14)	0.0357 (4)	
C3	0.4215 (3)	−0.1652 (3)	0.40281 (17)	0.0499 (5)	
H3	0.5420	−0.2152	0.3977	0.067 (8)*	
C4	0.3658 (4)	−0.1025 (3)	0.48919 (17)	0.0561 (6)	
H4	0.4487	−0.1092	0.5448	0.077 (9)*	
C5	0.1956 (4)	−0.0304 (3)	0.49927 (14)	0.0501 (6)	
H5	0.1605	0.0144	0.5607	0.050 (7)*	
C6	0.0752 (3)	−0.0228 (2)	0.42020 (13)	0.0379 (4)	
C7	0.0046 (3)	−0.06772 (19)	0.24441 (13)	0.0324 (4)	
C8	0.4849 (4)	−0.3237 (3)	0.2261 (2)	0.0635 (7)	
H8A	0.609	−0.2823	0.2415	0.095*	
H8B	0.486	−0.364	0.1600	0.095*	
H8C	0.4545	−0.4040	0.2722	0.095*	
C9	−0.1665 (5)	0.1023 (4)	0.5097 (2)	0.0737 (9)	
H9A	−0.088	0.186	0.5271	0.111*	
H9B	−0.163	0.0284	0.5603	0.111*	
H9C	−0.293	0.136	0.5010	0.111*	

### Atomic displacement parameters (Å^2^)

**Table:**

	*U*^11^	*U*^22^	*U*^33^	*U*^12^	*U*^13^	*U*^23^
O1	0.0636 (10)	0.0332 (7)	0.0383 (7)	0.0092 (7)	−0.0096 (7)	0.0031 (6)
O2	0.0596 (9)	0.0361 (8)	0.0508 (8)	0.0029 (7)	−0.0232 (8)	−0.0080 (7)
O3	0.0458 (8)	0.0453 (8)	0.0458 (8)	0.0111 (7)	0.0029 (7)	−0.0009 (7)
O4	0.0604 (10)	0.0590 (9)	0.0374 (7)	0.0120 (8)	0.0070 (7)	−0.0056 (7)
C1	0.0411 (9)	0.0241 (7)	0.0287 (7)	−0.0036 (7)	−0.0042 (7)	0.0020 (6)
C2	0.0387 (9)	0.0293 (8)	0.0392 (9)	−0.0036 (7)	−0.0016 (8)	0.0048 (7)
C3	0.0438 (11)	0.0493 (11)	0.0566 (12)	−0.0013 (10)	−0.0146 (10)	0.0096 (10)
C4	0.0653 (15)	0.0587 (13)	0.0442 (11)	−0.0136 (13)	−0.0232 (11)	0.0081 (11)
C5	0.0726 (16)	0.0481 (11)	0.0297 (9)	−0.0132 (11)	−0.0051 (10)	0.0000 (9)
C6	0.0495 (11)	0.0309 (8)	0.0333 (8)	−0.0061 (8)	0.0014 (8)	0.0026 (7)
C7	0.0379 (8)	0.0299 (8)	0.0295 (7)	0.0028 (7)	−0.0014 (7)	0.0002 (7)
C8	0.0588 (14)	0.0612 (15)	0.0704 (16)	0.0217 (13)	0.0115 (14)	0.0042 (14)
C9	0.083 (2)	0.0840 (19)	0.0544 (14)	0.0126 (18)	0.0152 (15)	−0.0216 (14)

### Geometric parameters (Å, °)

**Table:**

O1—C7	1.211 (2)	C3—H3	0.9700
O2—C7	1.314 (2)	C4—C5	1.379 (4)
O2—H2	0.87 (4)	C4—H4	0.9700
O3—C2	1.364 (2)	C5—C6	1.389 (3)
O3—C8	1.429 (3)	C5—H5	0.9700
O4—C6	1.361 (3)	C8—H8A	0.9819
O4—C9	1.424 (3)	C8—H8B	0.9819
C1—C2	1.395 (3)	C8—H8C	0.9819
C1—C6	1.403 (2)	C9—H9A	0.9607
C1—C7	1.497 (2)	C9—H9B	0.9607
C2—C3	1.388 (3)	C9—H9C	0.9607
C3—C4	1.375 (4)		
			
C7—O2—H2	114 (2)	O4—C6—C5	125.03 (19)
C2—O3—C8	117.56 (18)	O4—C6—C1	114.99 (17)
C6—O4—C9	118.6 (2)	C5—C6—C1	120.0 (2)
C2—C1—C6	119.51 (17)	O1—C7—O2	118.91 (17)
C2—C1—C7	120.69 (16)	O1—C7—C1	122.81 (16)
C6—C1—C7	119.77 (17)	O2—C7—C1	118.28 (15)
O3—C2—C3	124.32 (19)	O3—C8—H8A	109.5
O3—C2—C1	115.42 (16)	O3—C8—H8B	109.5
C3—C2—C1	120.20 (18)	H8A—C8—H8B	109.5
C4—C3—C2	119.2 (2)	O3—C8—H8C	109.5
C4—C3—H3	120.4	H8A—C8—H8C	109.5
C2—C3—H3	120.4	H8B—C8—H8C	109.5
C3—C4—C5	122.1 (2)	O4—C9—H9A	109.5
C3—C4—H4	119.0	O4—C9—H9B	109.5
C5—C4—H4	119.0	H9A—C9—H9B	109.5
C4—C5—C6	119.1 (2)	O4—C9—H9C	109.5
C4—C5—H5	120.5	H9A—C9—H9C	109.5
C6—C5—H5	120.5	H9B—C9—H9C	109.5
			
C8—O3—C2—C3	23.9 (3)	C9—O4—C6—C1	179.9 (2)
C8—O3—C2—C1	−159.0 (2)	C4—C5—C6—O4	176.4 (2)
C6—C1—C2—O3	−177.98 (16)	C4—C5—C6—C1	−1.9 (3)
C7—C1—C2—O3	0.1 (2)	C2—C1—C6—O4	−176.68 (16)
C6—C1—C2—C3	−0.7 (3)	C7—C1—C6—O4	5.2 (2)
C7—C1—C2—C3	177.33 (18)	C2—C1—C6—C5	1.7 (3)
O3—C2—C3—C4	176.9 (2)	C7—C1—C6—C5	−176.37 (17)
C1—C2—C3—C4	−0.1 (3)	C2—C1—C7—O1	−122.8 (2)
C2—C3—C4—C5	−0.1 (4)	C6—C1—C7—O1	55.3 (3)
C3—C4—C5—C6	1.0 (4)	C2—C1—C7—O2	57.4 (3)
C9—O4—C6—C5	1.6 (3)	C6—C1—C7—O2	−124.5 (2)

### Hydrogen-bond geometry (Å, °)

**Table:**

*D*—H···*A*	*D*—H	H···*A*	*D*···*A*	*D*—H···*A*
O2—H2···O1^i^	0.87 (4)	1.82 (4)	2.681 (2)	172 (4)

Symmetry codes: (i) −*x*, *y*−1/2, −*z*+1/2.
